# 

**Published:** 1899-12

**Authors:** 


CONTENTS.

| | PAGE |
|---|---|
| **Medical Bristol in the Eighteenth Century.** By W. H. HARSANT, F.R.C.S. Eng. | 297 |
| **The Varieties o Uterine Neoplasms and their Relative Frequency.** By W. ROGER WILLIAMS, F.R.C.S. Eng. | 314 |
| **A Case of Neuritic Muscular Atrophy ("Peroneal" Tpye).** By J. E. SHAW, M.B. Ed. | 319 |
| **Irreducible Intussusception: with Notes on a Fatal Case.** By G. L. KERR PRINGLE, M.D. Ed. | 322 |
| **Progress of the Medical Sciences:** | |
| **Medicine.** J. MICHELL CLARKE, M.D. | 326 |
| **Surgery.** W. M. BARCLAY | 331 |
| **Progress of the Medical Sciences:** | |
| **Laryngology and Rhinology.** BARCLAY J. BARON, M.B. | 336 |
| **Therapeutics.** BERTRAM M. H. ROGERS, M.D. | 339 |
| **Reviews of Books** | 342 |
| **Notes on Preparations for the Sick** | 366 |
| **Meetings of Sccieties** | 367 |
| **The Library of the Bristol Medico-Chirurgical Society** | 370 |
| **Local Medical Notes** | 374 |
| **The College of Physicians of Philadelphia** | 381 |
| **Scraps** | 381 |

